# Novel Teixobactin Analogues Show Promising In Vitro Activity on Biofilm Formation by *Staphylococcus aureus* and *Enterococcus faecalis*

**DOI:** 10.1007/s00284-024-03857-9

**Published:** 2024-09-10

**Authors:** Ahmed M. Amer, Colin Charnock, Sanko Nguyen

**Affiliations:** Department of Life Sciences and Health, Oslo Metropolitan University (OsloMet), Pilestredet 50, 0167 Oslo, Norway

## Abstract

The treatment of infections caused by biofilm-forming organisms is challenging. The newly discovered antibiotic teixobactin shows activity against a wide range of biofilm-forming bacteria. However, the laborious and low-yield chemical synthesis of teixobactin complicates its further development for clinical application. The use of more easily synthesized teixobactin analogues may offer promise in this regard. In this article, three newly developed analogues were tested for efficacy against *Staphylococcus aureus* and *Enterococcus faecalis*. Minimum inhibitory and -bactericidal concentrations were investigated. MIC values for *S. aureus* and *E. faecalis* ranged from 0.5–2 and 2–4 μg/mL, respectively. Moreover, the ability of the analogues to prevent biofilm formation and to inactivate bacterial cells in already established *S. aureus* biofilm on medical grade materials (PVC and PTFE) used in the production of infusion tubing and catheters were also tested. The analogues showed an ability to prevent biofilm formation and inactivate bacterial cells in established biofilms at concentrations as low as 1–2 μg/mL. Confocal laser scanning microscopy showed that the most promising analogue (TB3) inactivated *S. aureus* cells in a preformed biofilm and gave a reduction in biovolume. The relative ease of synthesis of the analogues and their in vitro efficacy, makes them promising candidates for pharmaceutical development.

**Graphical Abstract:**

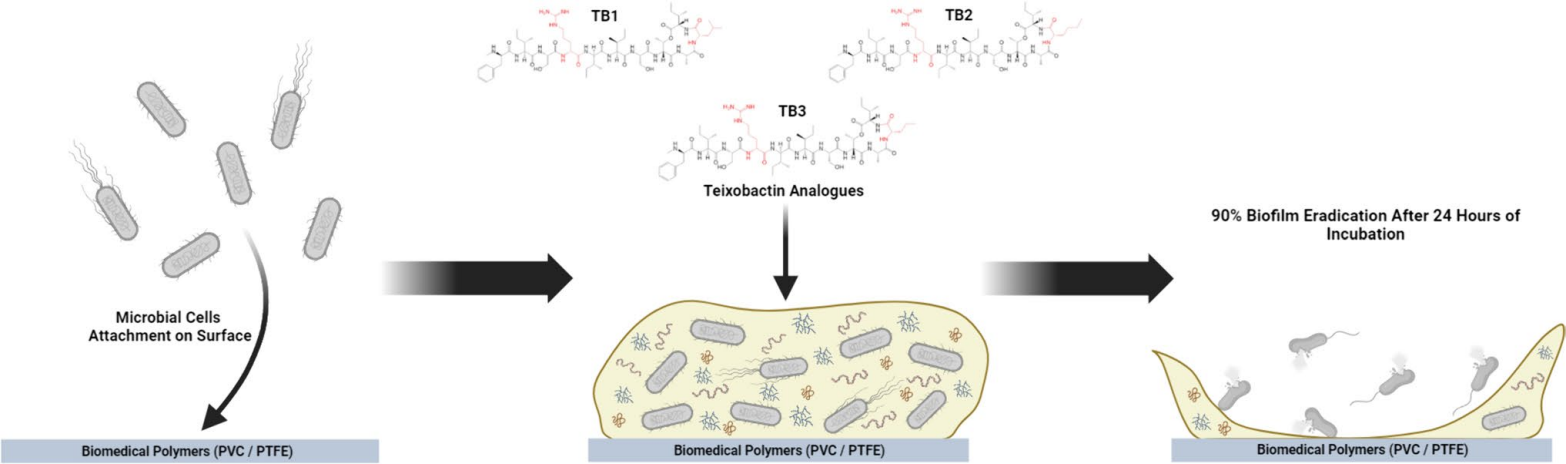

**Supplementary Information:**

The online version contains supplementary material available at 10.1007/s00284-024-03857-9.

## Introduction

Biofilms are the main cause of approximately 80% of recurrent and chronic microbial infections [1]. They can be either associated with the use of medical devices or can be formed on tissues resulting in various types of chronic infections [2]. The tolerance of biofilms for antibiotics can be attributed to among other factors, limited drug diffusion through the extracellular polymeric substances (EPS) and to cell conversion from the planktonic to dormant state with altered metabolic activities [2, 3]. The release of EPS and formation of the biofilm matrix is a fundamental step in the process of biofilm development. It protects the cells from the surrounding environment (e.g. components of the immune system and antibiotics) and provides mechanical and structural support. The EPS mostly consists of proteins, polysaccharides, extracellular DNA and lipids, all of which have a wide range of functions including adhesion, water retention, structural integrity and enzymatic activity [4]. In addition to being a physical barrier to antibiotic penetration, the biofilm matrix also plays a role in antibiotic degradation [5]. Bacterial cells inside the biofilm have in some cases been shown to be 1000 times more tolerant to antibiotics than planktonic cells [5].

Biofilm infections notably contribute to the emergence of antimicrobial resistance (AMR) through the transfer of resistance genes on mobile genetic elements, such as plasmids, inside the biofilm matrix [6]. Furthermore, biofilm formation is often associated with the development of hypermutator phenotypes of bacteria, which can show up to 1000-fold increases in mutation rates [1]. Mortality relating to AMR in 2019 was estimated at 1.27 million deaths based on data gathered from 204 countries [7]. Numbers are estimated to reach 10 million annually by 2050 if no firm actions are taken to resolve the problem [8, 9]. Unfortunately, the development of new antibiotics is being outpaced by the rapid increase of AMR. Over the past 40 years, most of the developed antibiotics were derived from antimicrobial agents already in clinical use. No antibiotics with novel modes of action have made it to the market since lipopeptide antibiotics emerged in the 1980s [10, 11].

In 2015, a novel class of antibiotics represented by the agent teixobactin was discovered. Teixobactin is produced by a newly discovered species provisionally named *Eleftheria terrae* [12]. Teixobactin is believed to exert its antimicrobial activity primarily by targeting lipid II and lipid III which are precursors for cell wall peptidoglycan and teichoic acid, respectively. The effect is generally limited to inhibition of cell wall synthesis in gram-positive bacteria, as the outer membrane of gram-negative bacteria prevents teixobactin penetration. Teixobactin demonstrated in vitro antimicrobial activity against multidrug resistant (MDR) bacteria, including methicillin-resistant *Staphylococcus aureus* (MRSA) and vancomycin-resistant *Enterococci* (VRE) [12]. Despite its potential as a new drug candidate, the chemical synthesis of teixobactin is challenging due to the presence of L-allo-enduracididine amino acid in the macrocyclic depsipeptide structure of teixobactin [13]. Methods for L-allo-enduracididine synthesis are multistep, laborious and with a relatively low yield. Therefore, new teixobactin analogues have been synthesised mainly by replacing L-allo-enduracididine with common and commercially available amino acids [14]. Structure–activity relationships (SAR) studies revealed that L-allo-enduracididine substitution by non-polar leucine at position 10 maintained significant antibacterial activity [13]. However, the substitution resulted in increased structural hydrophobicity, which is a common barrier to drug formulation and pharmaceutical development [13, 15]. To restore the hydrophilic/hydrophobic profile of the natural teixobactin molecule, a teixobactin analogue (D-Arg4-Leu10-teixobactin) was synthesised by replacing glutamine at position 4 with the cationic amino acid arginine [15]. This new, synthetic analogue demonstrated promising antimicrobial activity against both MRSA and VRE strains [15].

**Fig. 1:**
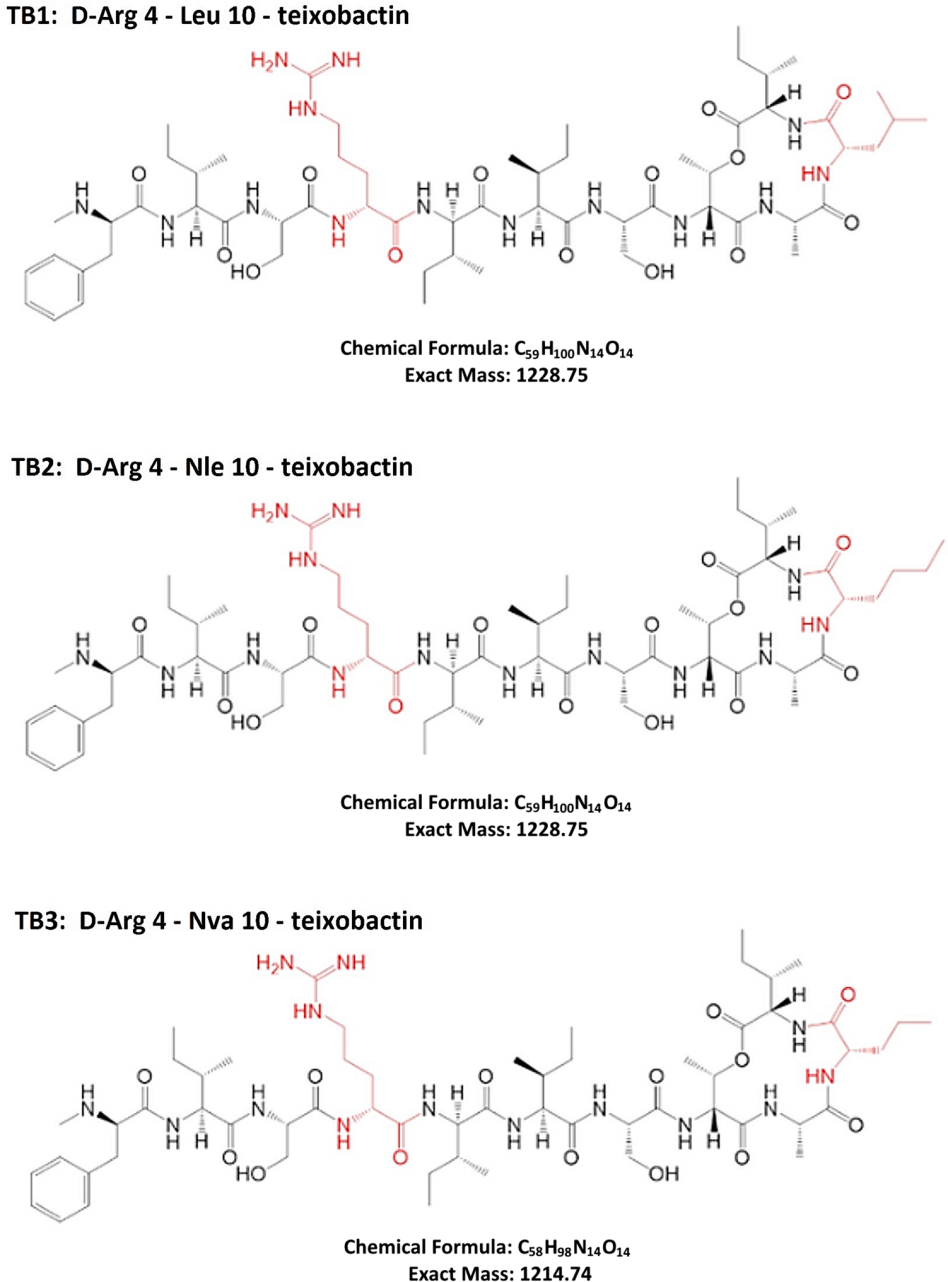
Chemical structures of the three teixobactin analogues (TB1, TB2 and TB3) investigated in this study. Red colour shows the substituted positions from the natural teixobactin structure. The L-allo-enduracididine amino acid residue at position 10 is replaced by an amino acid indicated by a three-letter code; *Leu* leucine, *Nle* norleucine, *Nva* norvaline. In all three analogues, the glutamine residue at position 4 is replaced by arginine (Arg)

In the present study, the antimicrobial and antibiofilm activity of D-Arg4-Leu10-teixobactin along with two structurally related novel analogues, D-Arg4-Nle10-teixobactin and D-Arg4-Nva10-teixobactin (Fig. 1), on the clinically important species *S. aureus* and *Enterococcus faecalis* were investigated. To our knowledge, this is the first study looking at the effect of teixobactin analogues on biofilm formation and treatment of mature biofilms on biomedical polymers used for production of medical devices and implants.

## Materials and Methods

### Materials

*S. aureus* (ATCC 29213) and *E. faecalis* (ATCC 29212) were purchased from American Type Culture Collection (Rockville, MD, USA). *S. aureus* clinical isolates P14 and P20 and *E. faecalis* clinical isolate P40 were isolated in our laboratory from the eyes of patients diagnosed with severe dry eye disease [16]. As previously described [16], the isolates were collected from the lower conjunctival sac and grown on a range of selective and non-selective agars. *E. faecalis* was identified after growing the isolate on *Enterococcus* selective agar and by partial sequencing of 16S rRNA. *S. aureus* was identified based on DNase production and latex slide agglutination (detecting clumping factor and protein A) in addition to 16S rRNA gene sequencing [16]. Additionally, the identity of the isolates was confirmed by whole genome sequencing. Protocols for 16S RNA and whole-genome sequencing are available in the supplementary data (Supplementary file 2). Cation-adjusted Mueller Hinton broth (MHB), tryptic soy broth (TSB), tryptic soy agar (TSA) and maximum recovery diluent (MRD) were purchased from Oxoid/Thermo Fischer (MA, USA). Vancomycin HCl (Product No. 94747), triphenyl tetrazolium chloride (TTC), crystal violet (CV) (ACS reagent C6158), Tween 80, dimethyl sulfoxide (DMSO) and phosphate buffered saline (PBS) tablets were purchased from Merck (Germany). FilmTracer™ LIVE/DEAD^®^ Biofilm Viability Kit was purchased from Invitrogen (USA). Natural teixobactin was provided by Novobiotic Pharmaceuticals (Cambridge, MA, USA). Teixobactin analogues were synthesized and provided by Dr. Ishwar Singh at Institute of Translational Medicine, University of Liverpool, as previously described [15]. All analogues were ≥ 90% pure as determined by HPLC–MS.

### Determination of Minimum Inhibitory Concentration (MIC) and Minimum Bactericidal Concentration (MBC)

MIC was determined using the broth microdilution method. Briefly, a stock solution 1600 μg/mL in DMSO of each antimicrobial compound was prepared. Vancomycin and natural teixobactin were used as quality controls. Serial dilutions were done in DMSO according to Clinical and Laboratory Standards Institute (CLSI) guidelines [17] to yield concentrations ranging from 1600 to 3.125 μg/mL. Each concentration was further diluted with MHB + 0.002% Tween 80 (1:100) to obtain final concentrations ranging from 16 to 0.03 μg/mL. One hundred μL of each final concentration was added to each well to round-bottom polystyrene and polypropylene 96-well microtiter plates (Corning^®^, USA) to investigate if plastic type affects the MIC results. Bacterial inoculums of *S. aureus* and *E. faecalis* were made from overnight cultures on TSA grown aerobically at 37 °C by suspending well-isolated colonies in 0.9% NaCl until 0.5 McFarland (approximately 10^8^ CFU/mL) was obtained spectrophotometrically. Suspensions were then diluted with cation-adjusted MHB + 0.002% Tween 80 such that a final concentration of 10^5^ CFU/mL was achieved in wells after inoculation [17]. As a negative control, some wells with growth medium were not inoculated with bacteria, while other wells were inoculated with bacteria and without any antibiotic as a positive control. Plates were incubated overnight at 37 °C and the MICs were determined visually by two researchers following the CLSI guidelines. After MIC determination, 100 μL from all wells containing TB analogues without visual bacterial growth (*S. aureus* ATCC 29213 and *E. faecalis* ATCC 29212) was spread out on TSA plates and incubated overnight at 37 °C. The number of colonies was counted to determine MBC, which is defined as the lowest antimicrobial concentration that kills ≥ 99.9% of the microorganism in the inoculum according to CLSI guidelines [18]. Experiments were repeated three times on separate occasions as independent experimental replicates.

### Effect of Teixobactin Analogues on Planktonic Growth

Inoculums of *S. aureus* (ATCC 29213) and *E. faecalis* (ATCC 29212) were prepared by suspending well-isolated colonies in 0.9% NaCl until 0.5 McFarland was obtained, followed by dilution with growth medium TSB + 1% glucose to a final concentration of 10^5^ CFU/mL. Teixobactin analogues or vancomycin were added in concentrations equal to ½ × MIC, MIC and 2 × MIC, and 2 mL of each mix was pipetted in into 24-well plates with a clear flat bottom (VisiPlates-24, PerkinElmer^®^, USA). Broth cultures without teixobactin or without cells were used as positive and negative growth controls, respectively. The optical density (OD) of the growth curves was measured with a multimode plate reader (Victor^3^, Perkin Elmer^®^, USA) at 590 nm every 15 min for 16 h at 37 °C. The curves were also plotted on a semi-logarithmic scale to determine the onset of the exponential growth phase and to calculate the culture doubling time (DT) as previously described [19]. The experiment was performed twice on separate occasions as independent experimental replicates.

### Inhibition of Biofilm Formation

Inoculums of *S. aureus* (ATCC 29213) and *E. faecalis* (ATCC 29212) were prepared as described in “Effect of Teixobactin Analogues on Planktonic Growth” section. Teixobactin analogues or vancomycin were added to bacterial suspensions in concentrations equal to ½ × MIC, MIC, 2 × MIC and 4 × MIC. Two hundred µl from each concentration for each mixture was transferred to wells (*n* = 9) in a 96-well polypropylene plate and incubated statically overnight at 37 °C to allow biofilm formation. Ten wells were incubated without antibiotic as positive controls. After incubation, wells were examined visually for bacterial growth and scored from 0 to + 4, where 0 presents no growth and + 4 is the maximum growth obtained with the positive control. Wells containing teixobactin analogues with significant visible growth reduction compared to the control were further assessed using three different methods i.e., colony counting, crystal violet (CV) assay and activity staining. The biofilm inhibitory concentration (BIC_90_), which is the lowest teixobactin analogue concentration inhibiting biofilm formation by > 90%, was determined using the results obtained by the different analysis methods. Prior to testing, planktonic cells were carefully removed by pipetting out the contents of each well and gently washed three times with PBS. The experiment was performed twice on separate occasions as independent experimental replicates.

#### Colony Counting

The biofilm was suspended into 0.1 mL MRD + 0.01% Tween 80 by scraping the wells’ inner wall using a sterile dental micro applicator brush (Guangzhou Jaan Medical, China). The contents were transferred to a microtube containing sterile 0.1 mm glass beads. To ensure maximal biofilm recovery, the same procedure was repeated twice for each well to obtain a final volume of 0.3 mL using the same brush and pipette tip. Following this, the tubes were sonicated (Elmasonic S30, Elma Ultrasonic Technology, Germany) for 5 min and vortexed for 3 cycles of 30 s with intermittent cooling to homogenize biofilms and release cells prior to plating. The suspensions were then serially diluted in MRD, plated on TSA, and incubated overnight at 37 °C. After incubation, the number of colonies was recorded and the percentage reduction in cell viability was calculated compared to the control.

#### Crystal Violet Assay

The assay was performed as previously described [20–22], with slight modifications: 0.1% w/v CV in purified water was prepared and filtered (0.22 µm) to remove any particles. Two hundred µl of the CV solution was added carefully to each well without disrupting the biofilm and the plate was incubated statically at 37 °C for 2.5 h. After incubation, the CV solution was removed, wells were washed carefully three times with PBS and 250 µl of 33% acetic acid was added to extract the colour from the biofilm by orbital shaking (Genie Temp-Shaker 100, Scientific Industries, USA) at 80 rpm for 15 min. Following extraction, 180 µl from each well was transferred to a flat-bottomed polystyrene 96-well plate and colour intensity was measured spectrophotometrically at 595 nm using the same plate reader (“Effect of Teixobactin Analogues on Planktonic Growth” section). The percentage reduction in biofilm formation was calculated compared to control wells using the following equation:

1 $$\%\; \text{Reduction}= [(\text{control absorbance}-\text{test absorbance})/\text{control absorbance}] \times 100$$

#### Activity Staining

The test was performed as previously described [23, 24], with slight modifications: 200 µL of 0.05% w/v triphenyl tetrazolium chloride (TTC) in TSB + 1% glucose was pipetted carefully into each well and incubated statically at 37 °C for 2.5 h. After incubation, the solution was removed, wells were washed three times with PBS and 250 µL of methanol was added to extract triphenyl formazan (TPF) colour from the biofilm by orbital shaking at 80 rpm for 15 min. Finally, 180 µL from each well was transferred to a new flat-bottomed polystyrene 96-well plate and colour intensity was measured spectrophotometrically at 490 nm. Percentage reduction in biofilm formation was calculated according to Eq. (1).

### Inactivation of Bacteria in Established Biofilms

Two structurally different materials used in biomedical applications were tested in this study i.e., polytetrafluoroethylene (PTFE for catheters production, Zeus Company, USA) and polyvinyl chloride (PVC for intravenous infusion tubing production, B. Braun, Germany). One cm rods of each type of plastic were disinfected by incubation at 70 °C in sterile water for 30 min followed by washing one time with 70% ethanol and three times with sterile water. An inoculum of *S. aureus* was prepared in TSB + 1% glucose as previously described (“Effect of Teixobactin Analogues on Planktonic Growth” section). Biofilm was allowed to grow on the rods for three days at 37 °C with gentle orbital shaking at 80 rpm. Fresh medium was replaced every day without disrupting the biofilm. After three days, the plastic pieces were washed three times with fresh PBS to remove planktonic cells and transferred to 6-well polystyrene flat bottom plates (Corning^®^, USA). To each well, 5 mL of cation-adjusted MHB containing ½ × MIC, MIC, 2 × MIC, 4 × MIC and 16 × MIC of teixobactin analogues or vancomycin were added to cover the rods. Plates were incubated for 24 h at 37 °C with orbital shaking at 80 rpm. Three plastic rods of each material were incubated without antimicrobial agents as positive controls, while another three of each material were incubated without cells as negative controls. After incubation, rods were washed three times with PBS. The experiment was performed twice on separate occasions as independent experimental replicates. The extent of inactivation of biofilm bacteria was assessed quantitatively by colony counting and qualitatively by activity staining as described below:

#### Colony Counting

Each rod was placed in a tube containing 1 mL MRD-broth + 0.01% Tween 80 and sterile 0.1 mm glass beads. The biofilm was then dispersed to free cells and plated on TSA using the same procedure described in “Colony Counting” section. After incubation, the number of colonies were recorded, and biofilm eradication concentration (BEC) was calculated as percentage reduction in CFU/mL relative to the control.

#### Activity Staining

Plastic rods were placed individually in wells of 6-well plates and covered with 5 mL of 0.05% w/v TTC solution in TSB + 1% glucose and incubated at 37 °C with orbital shaking at 80 rpm for 2.5 h. After incubation, rods were transferred to clean wells and colour intensity was graded visually using a scoring system, where (−) represents no growth and (+++) is the maximum growth. The colour intensity scoring was performed separately by two researchers to ensure fidelity in the interpretation.

### Confocal Laser Scanning Microscopy (CLSM) Analysis of Biofilms

An inoculum of *S. aureus* (ATCC 29213) was prepared by suspending well-isolated colonies in 0.9% NaCl until 0.5 McFarland was obtained, and the suspension was then diluted with TSB + 1% glucose to a final concentration of 10^5^ CFU/mL. Using 4-well chamber slides (Nunc Lab-Tek II CC2, Thermo Scientific), 1 mL of bacterial suspension was added to each chamber and allowed to grow for 72 h at 37 °C with shaking at 60 rpm. The medium was replaced carefully each day without disrupting the biofilm. After incubation, spent medium was replaced with 1 mL fresh medium containing 1, 2, 4 or 8 µg/mL of teixobactin analogue TB3 (*n* = 3). Other wells were incubated with only medium as control. TB3 was incubated with biofilms at 37 °C using low speed agitation (shaking at 60 rpm) for 24 h. After incubation, medium was again removed, and wells were washed twice with sterile water followed by addition of 200 μL of FilmTracer™ LIVE/DEAD^®^ Biofilm Viability Kit working solution in each well. The working solution was prepared by adding 3 μL of SYTO 9 stain and 3 μL of propidium iodide stain to 1 ml of sterile water. Slides were incubated for 30 min at room temperature protected from light. Afterwards, staining solution was removed, and chambers were washed twice with sterile water. Finally, 50 µL of antifading mounting solution (AFR3, Citifluor Ltd, UK) was added to each well and sealed beneath a coverslip. The slides were examined using a confocal microscope (TCS SP8 STED, Leica Microsystems, Germany). Fluorescence intensity measurements and 3D structures visualisation of the biofilms were generated from z-stacks by using ImageJ software (National Institutes of Health, Maryland, USA). Percentage cell viability was calculated after counting the number of live (green) and dead (red) cells in 5 randomly picked spots from each well. Biovolume for each biofilm was determined by firstly measuring the area covered by cells (live and dead) in all stacks after changing the images to binary (black/white) and determining the pixels which corresponds with bacterial cells using ImageJ software [25]. Afterwards, biovolume was calculated as reported by Heydorn et al. [26], which is defined as the pixels corresponding to biomass multiplied by voxel size and divided by substratum area of the image [26].

### Statistical Analysis

Results are presented as the mean ± standard deviation (SD). SD was calculated across all runs and replicates as the square root of the variance. The variance represents the data point’s deviation from the mean and calculated by taking the average of the squared differences between each data point and the mean. Significant difference between various treatments was determined using analysis of variance (ANOVA) and Bonferroni correction is used to adjust the significance level as multiple sets of results were involved in the comparisons. This was done by dividing the significance level (*P* value = 0.05) by the number of comparisons made. Whenever the SD value is not reported in the tables, it signifies that the experimental replicates yielded identical results with no observed variability.

### Accession Numbers of Clinical Isolates

The sequences of the clinical isolates used in this work have been deposited at the National Centre for Biotechnology Information (NCBI) repository under accession number SAMN40573642 for *E. faecalis* isolate P40 and accession numbers SAMN40573970 and SAMN40573973 for *S. aureus* isolates P14 and P20, respectively.

## Results

### Determination of MIC and MBC

The MIC values reported are the average obtained with polystyrene and polypropylene plates as no effect from the plate material on MIC was observed (Table 1). The measured MIC for natural teixobactin was similar to that previously reported by Ling et al. [12] for *S. aureus* ATCC 29213 (0.25 µg/mL). Furthermore, MIC values for vancomycin fell in the reported MIC range given in the CLSI guidelines for both *S. aureus* and *E. faecalis* [17], thus confirming the validity of the experimental approach. The newly developed teixobactin analogues MIC values were 1–2 and 2–4 μg/mL for *S. aureus* (ATCC 29213) and *E. faecalis* (ATCC 29212), respectively. The analogues demonstrated comparable microbiological activity on the clinical isolates of both bacterial strains as the MIC value for *S. aureus* clinical isolates ranged from 0.5 to 2 μg/mL and was found to be 2 μg/mL for the *E. faecalis* clinical isolate included in this study. The MIC of TB1 has previously been reported as < 0.0625 µg/mL against the same *S. aureus* strain (ATCC 29213) by Parmar et al. [15]. This disagreement might be due to the adoption of different test procedures. The MIC values reported by Parmar et al. were generally lower than those reported in the literature [27, 28].

**Table 1:** MIC (µg/mL) for *S. aureus* and *E. faecalis* with teixobactin analogues (TB1–3), natural teixobactin and vancomycin

	*S. aureus*			*E. faecalis*	
	ATCC 29213	P14	P20	ATCC 29212	P40
TB1	1 - 2	1	0.5 - 1	4	2
TB2	(1) - 2^a^	1 - 2	1 - 2	2 - (4)^a^	2
TB3	1 - 2	1	0.5 - 1	2 - (4)^a^	2
Teixobactin	0.25 - (1)	1	0.25	2 - 4	1 - 2
Vancomycin	1	ND	ND	2	ND

*ND* not determined

^a^The non-bracketed number occurred most often

To determine whether an antimicrobial compound is likely to be bactericidal or bacteriostatic, the MBC/MIC ratio is usually calculated. Generally, an MBC/MIC ratio ≤ 4 indicates bactericidal action [29]. MBC results for *S. aureus* ATCC 29213 and *E. faecalis* ATCC 29212 with all three analogues was found to be 8 µg/mL and > 16 µg/mL, respectively. No MBC values were determined for *E. faecalis* (ATCC 29212) at the concentrations investigated. However, since the MBC was > 16 µg/mL and the MIC was 2–4 µg/mL, the teixobactin analogues are bacteriostatic against the *E. faecalis* strain used in the present study as MBC/MIC ratio will be > 4.

### Effect of Teixobactin Analogues on Planktonic Growth

**Fig. 2:**
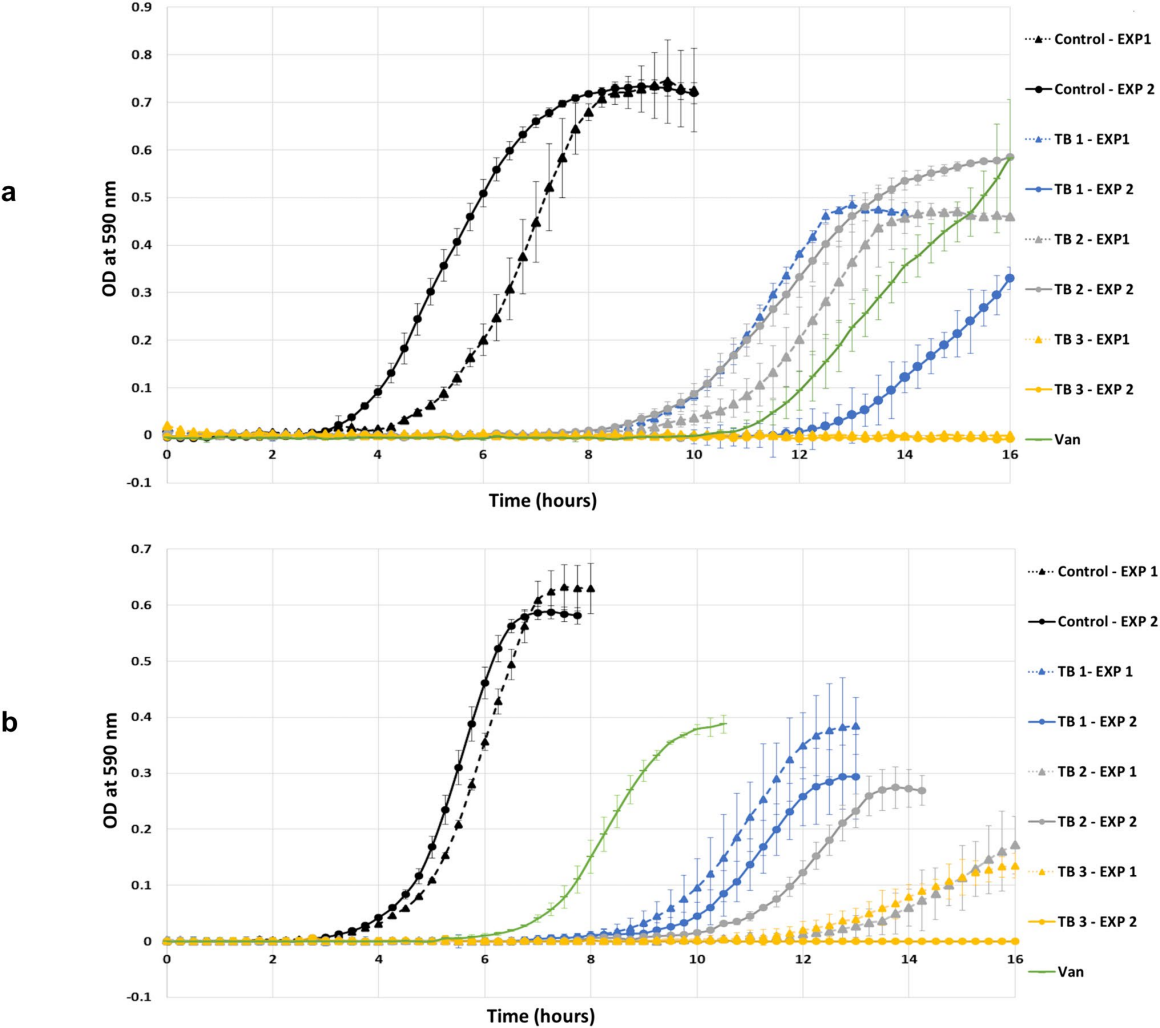
Growth curves of **a** *S. aureus* and **b** *E. faecalis* in the presence of ½ × MIC equivalent concentration of teixobactin analogues and vancomycin (Van) presented as mean ± SD

As MIC values are endpoint measurements, growth curves were generated to gain information on the activity of the analogues during different phases of culture development. The same medium was used to study both growth curves and grow biofilms. The obtained growth curves (Fig. 2) were in accordance with the MIC results as no growth was observed in concentrations ≥ MIC. At ½ × MIC, a measurable delay in the onset of the exponential phase was observed for both *S. aureus* and *E. faecalis* for all three teixobactin analogues (Table 2). The doubling time (DT) is a variable that can be used as a measurement of bacterial fitness. The ratio of increase in DT for each analogue compared to the control is given in Table 2. The period before the onset of the exponential phase was greatest with TB3 for both *S. aureus* and *E. faecalis*. In all but one experiment (experiment 1 with *E. faecalis*, Fig. 2), the onset of the exponential phase exceeded 16 h. By comparison, the time to onset of exponential growth ranged from 8 to 12 h with TB1 and TB2. From the calculated values for the doubling time (DT) presented in Table 2, the DT for *E. faecalis* had an average increase of 11% and 42% with TB1 and TB2, respectively, while with *S. aureus* the increase was notably higher at 56% and 92%, respectively for the same analogues. Statistical analysis of the results demonstrated significant delay in the exponential phase onset with teixobactin analogues compared to the control with both bacterial species. No significant differences were detected between the analogues tested. Moreover, the changes observed in the doubling time with the analogues were insignificant compared to the control. The results show that the analogues affect bacterial ability to adapt and grow even at ½ × MIC. The less profound effect of the teixobactin analogues on *E. faecalis* compared to *S. aureus* is in agreement with the MBC/MIC results.

**Table 2:** Time to onset of the exponential growth (log phase) and doubling times of *S. aureus* and *E. faecalis* without addition of any antibiotic (control) and after treatment with teixobactin analogues or vancomycin

	*S. aureus* ATCC 29213				*E. faecalis* ATCC 29212			
	Onset of exponential growth (h)		Doubling time (h)		Onset of exponential growth (h)		Doubling time (h)	
	Exp 1	Exp 2	Exp 1	Exp 2	Exp 1	Exp 2	Exp 1	Exp 2
Control	3 ± 0.2	2.5 ± 0.3	0.42 ± 0.027	0.41 ± 0.017	3.25 ± 0.2	3.25 ± 0.1	0.56 ± 0.045	0.49 ± 0.029
TB1	8.25 ± 0.3	11.75 ± 0.1	0.60 ± 0.038 (1.43)^a^	0.69 ± 0.042 (1.68)^a^	8.25 ± 0.1	9 ± 0.5	0.62 ± 0.033 (1.12)^a^	0.61 ± 0.052 (1.09)^a^
TB2	8.25 ± 0.1	8 ± 0.2	0.74 ± 0.035 (1.76)^a^	0.85 ± 0.061 (2.07)^a^	12.25 ± 0.3	9.75 ± 0.4	0.91 ± 0.063 (1.63)^a^	0.67 ± 0.049 (1.20)^a^
TB3	ND	ND	ND	ND	11.75 ± 0.5	ND	0.84 ± 0.048 (1.50)^a^	ND
Vancomycin	10 ± 0.5		0.58 ± 0.016 (1.41)^a^		5.5 ± 0.2		0.54 ± 0.027 (1.10)^a^	

Exp 1 and Exp 2 denote the two separate experiments performed. Results shown as mean ± SD and significant difference was determined using analysis of variance (ANOVA) with Bonferroni correction (*P* value < 0.05)

^a^Bracketed numbers are the fold increase in doubling time compared to the control

*ND* not determined

### Inhibition of Biofilm Formation

The administration of prophylactic antibiotics and the use of antibiotic coated medical devices to prevent biofilm formation are among the currently implemented strategies for combating biofilms in the clinical setting [30, 31]. Therefore, testing the ability of antibiotics to prevent biofilm formation is pertinent. Biofilm experiments were performed in TSB + 1% glucose which is a commonly used growth medium for studies involving both *S. aureus* and *E. faecalis* biofilms [20–22, 32–35]. BIC_90_ was determined by three different methods which are among the most common measures of biofilm quantification [36]. Results are summarized in Table 3 as biofilm inhibitory concentration (BIC90) values in µg/mL and presented for each method in detail below.

**Table 3:** Summary of biofilm inhibitory concentration (BIC_90_) values in µg/mL (mean ± SD) for teixobactin analogues and vancomycin determined with three different methods

	*S. aureus* ATCC 29213			*E. faecalis* ATCC 29212		
	Colony counting	Crystal violet	Activity staining	Colony counting	Crystal violet	Activity staining
TB1	4	4	4	8	8	ND
TB2	4	4	4	8	8	ND
TB3	3 ± 1.41	3 ± 1.41	3 ± 1.41	4	4	ND
Vancomycin	1	1	1	2	> 8	ND

The absence of SD value signifies that the experimental replicates yielded identical results with no observed variability

*ND* not determined as no colour change was observed (see “Activity Staining” section)

#### Visual Scoring and Colony Counting

Regarding *S. aureus*, all analogues achieved greater than 90% reduction in the number of viable cells relative to the control at concentrations equal to 2 × MIC in both rounds of testing. TB3 even showed slightly higher activity by inhibiting biofilm formation at 1 × MIC in one round of testing. With respect to *E. faecalis*, similar results were obtained for TB1 and TB2. For TB3, no visual growth was observed at 1 × MIC equivalent concentration in two independent rounds of testing indicating higher antibiofilm activity than TB1 and TB2 (Supplementary file 1—Table S1). These results are in line with the growth curves observed for the teixobactin analogues (“Effect of Teixobactin Analogues on Planktonic Growth” section) suggesting the SAR effect of Nva substitution at position 10, which results in higher antimicrobial and antibiofilm effect with TB3.

#### Crystal Violet Assay

CV staining is relatively easy to perform, less expensive and less laborious than colony counting [36]. However, CV is non-selective for cells and it stains negatively charged biofilm matrix molecules (e.g. nucleic acids, polysaccharides and proteins) [37]. Thus, the results represent the whole biofilm mass including both cells and extracellular components. In the present study, the calculated percentage reduction in biofilm formation by CV staining was similar to that obtained with colony counting (Supplementary file 1—Table S2). However, *E. faecalis* results with vancomycin did not reflect the actual biofilm reduction observed by colony counting. The deviation could be attributed to vancomycin-induced production of one or more biofilm matrix components which can possibly bind to crystal violet. Such deviations can occur especially after stressful treatment of bacteria with antibiotics [37]. These findings support the importance of also including colony counting or similar direct measures of cell viability when analysing biofilms.

#### Activity Staining

The calculated reduction in TPF production compared to the control wells supports the colony counting and CV results for *S. aureus* (Supplementary file 1—Table S3). Results obtained with *E. faecalis* were inconclusive as no red colour development was observed even in the control wells without antibiotic treatment. The reason for this is not known but suggests a limitation of using TTC as a measure of biofilm production for this species. Biofilm formation may affect the cells metabolic activity, and this may have affected the assay outcomes.

### Inactivation of Bacteria in Established Biofilms

All three teixobactin analogues demonstrated a concentration-dependent effect on *S. aureus* biofilm eradication on both PVC and PTFE surfaces. Antibiotic concentrations resulting in 50% and 90% eradication of the biofilm, BEC_50_ and BEC_90_, respectively, are presented in Table 4. All analogues showed effective inactivation of bacterial cells in the biofilm at concentrations as low as the MIC (1 µg/mL). The activity of the analogues increased slightly in the following order TB3 > TB2 > TB1. Results from the activity staining method (Table 5) showed similar patterns to colony counting, where the activity slightly increased in the same order (TB3 > TB2 > TB1). Despite the low MIC value for vancomycin against *S. aureus* (1 µg/mL), BEC_90_ values of vancomycin were 4- and 16-folds higher than the MIC with PVC and PTFE, respectively.

**Table 4:** BEC_50_ and BEC_90_ of *S. aureus* in µg/mL (mean ± SD) after treatment of PVC and PTFE with teixobactin analogues and vancomycin

	PVC		PTFE	
	BEC_50_ (µg/mL)	BEC_90_ (µg/mL)	BEC_50_ (µg/mL)	BEC_90_ (µg/mL)
TB1	1.5 ± 0.71	5 ± 4.24	4	4
TB2	1.5 ± 0.71	2	2	4
TB3	1.5 ± 0.71	1	1.5 ± 0.71	3 ± 1.41
Vancomycin	4	4	4	16

The absence of SD value signifies that the experimental replicates yielded identical results with no observed variability

**Table 5:** Qualitative scoring of *S. aureus* biofilm based on triphenyl formazan colour intensity on PVC and PTFE after treatment with teixobactin analogues

Plastic type	PVC		PTFE	
Scoring scale				

Concentration in µg/mL (MIC equivalent)^a^	Recorded colour scores			
	Exp 1	Exp 2	Exp 1	Exp 2
Control	+++	+++	+	++
TB1				
1 (½ × MIC)	+	+	+	++
2 (1 × MIC)	++	+	+	+
4 (2 × MIC)	++	+	−	+
TB2				
1 (½ × MIC)	+	+	+	++
2 (1 × MIC)	++	+	−	++
4 (2 × MIC)	+	+	−	−
TB3				
1 (½ × MIC)	+	+	+	+
2 (1 × MIC)	+	+	−	+
4 (2 × MIC)	+	+	−	−

^a^At and above 8 µg/mL (4 × MIC) no colouration was observed

### Confocal Laser Scanning Microscopy (CLSM) Analysis of Biofilms

**Fig. 3:**
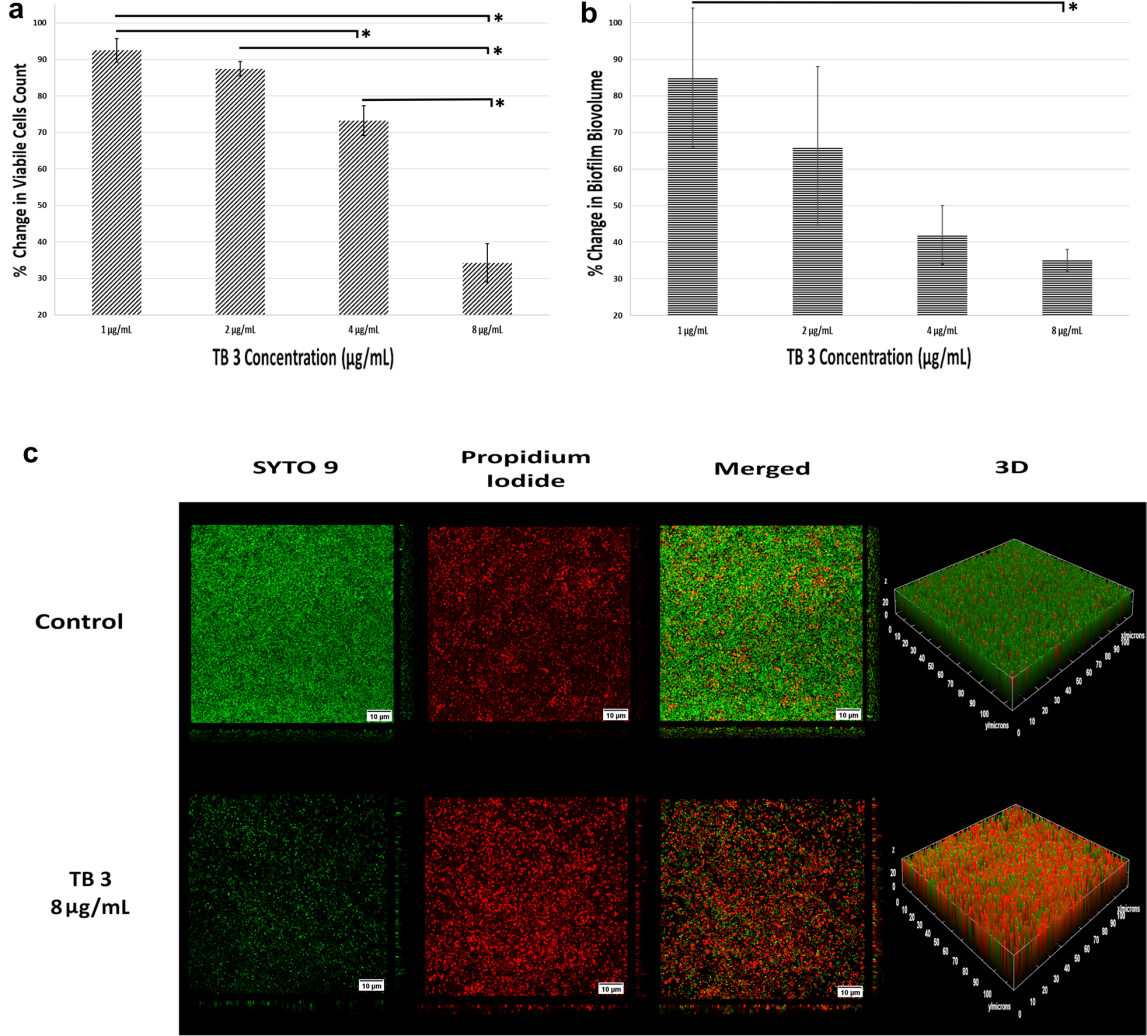
Effect of different concentrations of TB3 on **a** percentage bacterial cell viability presented as mean ± SD and **b** percentage change in biovolume compared to control presented as mean ± SD. **c** shows 2D images for live cells (SYTO 9), dead cells (propidium iodide) and 3D visualizations obtained from CLSM z-stacks for the control and 8 µg/mL of TB3. (*) indicates significant difference (*P* < 0.05)

The results obtained from CLSM images analysis after testing different concentrations of TB3 on a preformed *S. aureus* biofilm on glass chamber are presented in Fig. 3. With respect to biofilm formation and inactivation, bacterial cell viability is the most critical operational parameter, as it directly influences biofilm production. Bacterial cell viability was investigated using a combination of dyes where actively metabolizing cells stain green and dead cells stain red. Increasing the concentration of TB3 resulted in proportional significant decrease (*P* < 0.05) in *S. aureus* viability which was 34.2 ± 5.3% with the highest TB3 concentration tested (8 µg/mL). Additionally, biovolume was calculated to demonstrate the ability of TB3 to induce biofilm thinning and removal. Biovolume represents the overall volume occupied by the biofilm and is commonly used as a parameter in biofilm studies as it provides valuable information regarding the biofilm size and biomass [26, 38]. TB3 demonstrated the ability to significantly disrupt preformed biofilm after treatment for only 24 h. TB3 at 8 µg/mL concentration resulted in decreased biovolume by approximately 75% compared to the control.

## Discussion

Antibiotic resistance is a global health threat, prompting research aimed at the discovery and development of new agents with clinical potential. Here we show that three easily synthesized analogues of the natural antibiotic teixobactin, retain promising activity in vitro against the important pathogens *S. aureus* and *E. faecalis*. All three teixobactin analogues gave similar MIC values of 1–2 and 2–4 μg/mL for *S. aureus* (ATCC 29213) and *E. faecalis* (ATCC 29212), respectively. The activity also extended to the clinical isolates of both species. Although the MIC values for the teixobactin analogues were slightly higher compared to the ones reported for natural teixobactin (this study and [12]), the advantage of the analogues for future drug development, lies in their relative ease of production compared with the natural product. Despite structural differences, the teixobactin analogues seem to retain most of the bactericidal activity of the natural teixobactin against *S. aureus* [12]. For *S. aureus* strain tested in this work*,* the teixobactin analogues were bactericidal, which is likely due to the inhibition of peptidoglycan and teichoic acid synthesis leading to disruption of cell wall formation and cell lysis [39]. However, no bactericidal effect was detected against *E. faecalis*, which can probably be attributed to an *E. faecalis* intrinsic tolerance mechanism against antibiotics targeting cell wall [40]. This suggests a potentially restricted application of teixobactin and its analogues in treating infections caused by this species*.* To our knowledge, Gebhard et al*.* study [41] is the only report on MBC values of teixobactin or its analogues for *E. faecalis*. They reported MIC and MBC of teixobactin as 2 µg/mL and 16–32 µg/mL, respectively, which supports the findings of the current work. The same study also reported that vancomycin MBC for the same bacteria species was higher than 128 μg/mL. The authors attributed *E. faecalis* tolerance to teixobactin to a change in the CroRS two-component regulatory system. This change resulted in upregulation of the expression of major genes involved in cell wall biogenesis [41]. The same tolerance mechanism was also reported with vancomycin and bacitracin, indicating that it can be an intrinsic tolerance mechanism in *E. faecalis* against antibiotics targeting cell wall [40].

To our knowledge, details of the effect of sub-lethal concentrations of teixobactin on the development of bacterial populations in liquid culture (growth curves) has not previously been investigated. The observed prolongation of the lag phase in the presence of teixobactin analogues might be attributed to a defence mechanism that allows the bacteria to tolerate cellular stress induced by the antimicrobial agent. This effect has been described as the “tolerance by lag” phenomenon [42]. Bacterial growth responses are agent-specific as they are affected by the antimicrobial’s mechanism of action. Theophel et al*.* [43] reported that antibiotics affecting cell wall or membrane integrity (e.g. vancomycin and daptomycin) delay the onset of bacterial growth. Conversely, antibiotics affecting DNA replication or protein synthesis (e.g. moxifloxacin and gentamicin) show initial normal growth followed by induced alteration [43]. The observed delay in the onset of the exponential phase with both *S. aureus* and *E. faecalis* agrees with previously reported growth responses for antibiotics affecting cell envelope integrity [43, 44]. Additionally, it is noteworthy that even after the onset of the exponential growth, the maximum OD_590_ reached was substantially lower for wells containing antibiotics compared to the antibiotic-free control for both types of bacteria (Fig. 2). The reason for this observation is not yet clear, but it has been reported by others [43]. It is possible that the prolonged lag phase consumes energy or generates products inhibitory for later growth, but these remain speculations. Although the MIC values for the three teixobactin analogues (Leu in TB1, Nle in TB2 and Nva in TB3) were equal (Table 1), bacterial growth in the presence of TB3 was more severely restricted than in the presence of the other analogues. This suggests that the length of the hydrophobic side chain at position 10 critically affects the structure–activity relationship (SAR). Although requiring further investigation, the finding receives support from Jin et al*.* [28], where a onefold decrease in MIC for *S. aureus* was reported when norvaline (Nva) was introduced in position 10 of the teixobactin structure compared to norleucine (Nle) and isoleucine (Ile) substitutions [28]. The authors proposed that the higher microbiological activity is associated with the presence of a linear three-carbon side chain in Nva [28]. This substitution is also present in TB3 (Fig. 1).

Three approaches (colony counting, CV and activity staining) were employed to test the ability of the analogues to inhibit biofilm formation. Results of the three analysis methods were consistent and generated the same BIC_90_ values. BIC_90_ for *S. aureus* with TB1 and TB2 was found to be 4 µg/mL (2 × MIC). While TB3 demonstrated slightly higher activity with an average BIC_90_ of 3 µg/mL (Table 3). BIC_90_ values for *E. faecalis* were 2 × MIC (8 µg/mL) for both TB1 and TB2, while for TB3 the BIC_90_ was 1 × MIC (4 µg/mL). Although mutually supportive in analysis of biofilm formation, the three analytical approaches have individual weaknesses and strengths. The CV assay provides a measurement of total biofilm components (including cells and extracellular material). In contrast, colony counting and metabolic assays offer more selective measurements as they are not directly affected by extracellular material and essentially reflect the presence of viable cells. A particular advantage of activity staining is that viable cells may not form colonies on agar. Metabolically active cells will reduce the TTC substrate to TPF resulting in red colour development [23]. The consistent findings across all three methodologies strengthen the claim that teixobactin analogues possess the capability to prevent biofilm formation. Moreover, the increased efficacy observed with TB3 supports the proposed SAR.

The process of biofilm development on surfaces is multifactorial, depending on the bacterial strain, the surrounding environment and physicochemical properties of the material surface [45]. Given this background, we decided to investigate two structurally different materials used in the production of medical devices, PVC and PTFE, for biofilm formation. Antibiofilm activity on both materials varied between the different teixobactin analogues. Activity increased in the following order TB3 > TB2 > TB1. This is also in line with previously discussed SAR. Moreover, the agreement of activity staining results with colony counting, suggests the possibility of using activity staining as an easy and time-saving qualitative method for preliminary screening of antibiotic activity against biofilms. The difference between MIC and BEC_90_ with teixobactin analogues was much lower than that seen with vancomycin (e.g. only 2-folds with TB3 compared with 16-folds for vancomycin). This would suggest a better ability of teixobactin analogues to penetrate the biofilm matrix. This is highly interesting in terms of drug development, as insufficient antibiotic penetration is a main cause of treatment failure of biofilm based infections [2, 3]. Moreover, the higher hydrophobicity of teixobactin analogues compared to the water-soluble vancomycin may result in greater tendency of the analogues to adsorb onto plastic surfaces than vancomycin resulting in lower BEC_90_ values. In a published study investigating peptide interaction with hydrophobic fluorocarbon surfaces, peptide adsorption on the surface occurred within a few minutes of incubation through hydrophobic attractions between leucine residues and the material surface [46].

Teixobactin analogue (TB3), which exhibited the most promising effect against established biofilm, was chosen for further analysis using CLSM. Biofilm cell viability and biovolume demonstrated a proportional decrease corresponding to an increase in TB3 concentration from 1 to 8 µg/mL (½ × MIC to 4 × MIC). Three dimensional visualisations for the collected image stacks show a considerable change in the biofilm population after treatment for 24 h with 8 µg/mL of TB3, as the count of viable cells (depicted in green in Fig. 3) decreased significantly (*P* < 0.05) by 65% compared to the control wells, while the biovolume exhibited a reduction of approximately 75% relative to the control.

## Conclusion

All three novel teixobactin analogues proved to be effective at clinically relevant concentrations against the medically important, biofilm-forming species *S. aureus* and *E. faecalis*, including both culture-collection strains and some clinical isolates. The analogues were able to inhibit biofilm formation and demonstrated higher activity on pre-established *S. aureus* biofilms than vancomycin. This together with the relative ease of production of the analogues compared to the natural teixobactin makes them interesting candidates for further pharmaceutical development. The impact of position 10 substitution on SAR of teixobactin analogues was also confirmed: substitution with norvaline resulted in a notable reduction in the agent concentration needed to eradicate biofilms. Future work could include investigations on a wider range of bacteria including more clinical isolates and the development of appropriate pharmaceutical formulations. We are currently working on incorporating teixobactin analogues in liposomal carriers to see if antibacterial efficacy and drug stability can be improved.

## Supplementary Information

Below is the link to the electronic supplementary material.

Supplementary file1 (DOCX 21 KB)

Supplementary file2 (DOCX 17 KB)

## Data Availability

All data and material are available.
