# Supplementary material for: Novel Teixobactin Analogues Show Promising In Vitro Activity on Biofilm Formation by Staphylococcus aureus and Enterococcus faecalis
Source: Curr Microbiol. 2024 Sep 10;81(10):349. doi: 10.1007/s00284-024-03857-9 (PMC11387452; doi:10.1007/s00284-024-03857-9)
Supplement: Supplementary file 1 — Supplementary file1 (DOCX 21 KB) [file 284_2024_3857_MOESM1_ESM.docx]

**Supplementay material**

**Table S.1**

Visual scoring of growth intensity (in brackets) and percentage reduction in *S. aureus* and *E. faecalis* colonies after incubation for 24 hours with teixobactin analogues and vancomycin (Van). Exp 1 and exp 2 denote two independent rounds of testing.

|  | ***S. aureus* ATCC 29213** | | | | | | | | ***E. faecalis* ATCC 29212** | | | | | | | |
| --- | --- | --- | --- | --- | --- | --- | --- | --- | --- | --- | --- | --- | --- | --- | --- | --- |
|  | **½ X MIC** | | **MIC** | | **2X MIC** | | **4X MIC** | | **½ X MIC** | | **MIC** | | **2X MIC** | | **4X MIC** | |
|  | **Exp 1** | **Exp 2** | **Exp 1** | **Exp 2** | **Exp 1** | **Exp 2** | **Exp 1** | **Exp 2** | **Exp 1** | **Exp 2** | **Exp 1** | **Exp 2** | **Exp 1** | **Exp 2** | **Exp 1** | **Exp 2** |
| **TB1** | (4+) | (4+) | (4+) | (4+) | (0)  99.85% | (0)  98.15% | (0)  97.57% | (0)  99.99% | (3+) | (3+) | (2+) | (3+) | (0)  99.56% | (0)  99.14% | (0)  99.98% | (0)  99.97% |
| **TB2** | (4+) | (4+) | (4+) | (4+) | (0)  98.75% | (0)  97.22% | (0)  91.64% | (0)  99.99% | (3+) | (3+) | (2+) | (2+) | (0)  99.32% | (0)  99.20% | (0)  99.94% | (0)  99.99% |
| **TB3** | (4+) | (4+) | (3+) | (0)  99.87% | (0)  91.21% | (0)  99.87% | (0)  99.43% | (0)  99.99% | (3+) | (3+) | (0)  99.98% | (0)  99.88% | (0)  99.92% | (0)  99.99% | (0)  99.99% | (0)  99.99% |
| **Van** | (4+) | | (0)  99.47% | | (0)  99.96% | | (0)  99.99% | | (3+) | | (0)  92.70% | | (0)  99.97% | | (0)  99.99% | |

**Table S.2**

Percentage reduction in *S. aureus* and *E. faecalis* crystal violet absorbance after incubation for 24 hours with teixobactin analogues and vancomycin (Van). Exp 1 and exp 2 denote two independent rounds of testing.

|  | ***S. aureus* ATCC 29213** | | | | | | | | ***E. faecalis* ATCC 29212** | | | | | | | |
| --- | --- | --- | --- | --- | --- | --- | --- | --- | --- | --- | --- | --- | --- | --- | --- | --- |
|  | **½ X MIC** | | **MIC** | | **2X MIC** | | **4X MIC** | | **½ X MIC** | | **MIC** | | **2X MIC** | | **4X MIC** | |
|  | **Exp 1** | **Exp 2** | **Exp 1** | **Exp 2** | **Exp 1** | **Exp 2** | **Exp 1** | **Exp 2** | **Exp 1** | **Exp 2** | **Exp 1** | **Exp 2** | **Exp 1** | **Exp 2** | **Exp 1** | **Exp 2** |
| **TB1** | ND | ND | ND | ND | 96.08% | 95.44% | 99.06% | 99.02% | ND | ND | ND | ND | 92.54% | 100% | 100% | 100% |
| **TB2** | ND | ND | ND | ND | 97.04% | 98.16% | 100% | 95.90% | ND | ND | ND | ND | 82.34% | 94.51% | 100% | 100% |
| **TB3** | ND | ND | ND | 100% | 100% | 100% | 99.01% | 100% | ND | ND | 100% | 94.22% | 100% | 99.91% | 100% | 100% |
| **Van** | ND | | 97.10% | | 95.20% | | 99.14% | | ND | | 69.60% | | 72.35% | | 70.83% | |

* ND: Not determined (testing was only done for wells without detectable visual growth).

**Table S.3**

Percentage reduction in *S. aureus* activity stain absorbance after incubation for 24 hours with teixobactin analogues and vancomycin. Exp 1 and exp 2 denote two independent rounds of testing.

|  | ***S. aureus* ATCC 29213** | | | | | | | |
| --- | --- | --- | --- | --- | --- | --- | --- | --- |
|  | **½ X MIC** | | **MIC** | | **2X MIC** | | **4X MIC** | |
|  | **Exp 1** | **Exp 2** | **Exp 1** | **Exp 2** | **Exp 1** | **Exp 2** | **Exp 1** | **Exp 2** |
| **TB1** | ND | ND | ND | ND | 99.29% | 98.45% | 99.25% | 99.34% |
| **TB2** | ND | ND | ND | ND | 99.72% | 99.44% | 99.81% | 99.53% |
| **TB3** | ND | ND | ND | 100% | 98.40% | 100% | 99.44% | 99.15% |
| **Van** | ND | | 98.87% | | 97.74% | | 100% | |

* ND: Not determined (testing was only done for wells without detectable visual growth).
