# Supplementary material for: Novel Teixobactin Analogues Show Promising In Vitro Activity on Biofilm Formation by Staphylococcus aureus and Enterococcus faecalis
Source: Curr Microbiol. 2024 Sep 10;81(10):349. doi: 10.1007/s00284-024-03857-9 (PMC11387452; doi:10.1007/s00284-024-03857-9)
Supplement: Supplementary file 2 — Supplementary file2 (DOCX 17 KB) [file 284_2024_3857_MOESM2_ESM.docx]

**Identification of the clinical isolates**

**1. 16S rDNA amplification:**

The PCR reaction mixture (50 µL) contained 3 µL of 25 mM MgCl2 (Promega), 1 µL dNTPS 10 mM (Promega), 10 µL HotStart DNA polymerase buffer, 0.2 µL HotStart DNA polymerase 5 U/µL (Promega), 0.25 µL of each of primers 27f (AGA GTT TGA TCA TGG CTC A) and 1492r (TAC GGT TAC CTT GTT ACG ACT T) [100 µM stock of standard à la carte sequencing primers from MWG Eurofins] and PCR-grade water to 50 µL. To provide the template, a flame-sterilized steel pin was touched onto a bacterial colony and the pinpoint of material was transferred to the reaction mix. PCR conditions were: one cycle at 95 °C (10 min) followed by 32 cycles of 95 °C/45s, 52 °C/45 s and 72 °C/1 min. This was followed by a final elongation of 72 °C/12 min. PCR products were checked for purity by agarose electrophoresis and DNA concentrations were measured using Qubit™ dsDNA BR Assay Kit (Thermo Fisher Scientific, Waltham, MA, USA). PCR products were sequenced with Sanger sequencing on both strands at a commercial laboratory (Eurofins Genomics, Germany) using the PCR primers. Sequences were aligned using Clustal Omega [3], and the consensus region of overlap was used for purposes of identification. Sequences were compared to reposited sequences in the GenBank sequence database using BLAST (Basic Tool Alignment Search Tool) (https://blast.ncbi.nlm.nih.gov/Blast.cgi). The curated ‘Reference RNA sequence’ setting in BLAST was used to assign the 16S sequences to a named taxon.

**2. Whole genome sequencing**

Whole genome sequences of the clinical isolates are available under accession number SAMN40573642 for *E. faecalis* isolate P40 and accession numbers SAMN40573970 and SAMN40573973 for *S. aureus* isolates P14 and P20, respectively. The two isolates form part of a larger study involving WGS of multiple dry eye isolates which is currently being prepared for publication. More details of the methodology can be made available on request. In brief, genomic DNA was extracted from the bacterial isolates after inoculation on brain heart infusion agar (ThermoFisher Scientific, CM1136B) using the GenElute Bacterial Genomic DNA kit (NA2120, Sigma-Aldrich-Merck) according to the manufacturer’s instructions. DNA library preparation and sequencing were done by Eurofins Genomics (Germany). Briefly, DNA libraries with 150 base pair paired-end reads were generated through fragmentation, end-repair, A-tailing, adapter ligation, size selection and library amplification. Afterwards, sequencing was done by Illumina technology (NovaSeq6000, PE150 mode) and genome assembly and identification was performed by mapping the reads to reference genomes *S. aureus* NCTC 8532 and *E. faecalis* NCTC 775.
